# P22 Evaluating the impact of antimicrobial pharmacist and consultant microbiologist–led AMS ward rounds at York and Scarborough Teaching Hospital

**DOI:** 10.1093/jacamr/dlag102.028

**Published:** 2026-06-26

**Authors:** Ammara Asif, Barry Neish, Chris Beard, Dave Hamilton, Damian Mawer, Katrina Blackmore, Mathew Heaney, Heather Mackenzie, Paul Jackson, David Preece, Rachel Phillips, Rebecca Ince

**Affiliations:** Microbiology and Pharmacy Department, York and Scarborough Teaching Hospitals NHS Trust, UK; Microbiology and Pharmacy Department, York and Scarborough Teaching Hospitals NHS Trust, UK; Microbiology and Pharmacy Department, York and Scarborough Teaching Hospitals NHS Trust, UK; Microbiology and Pharmacy Department, York and Scarborough Teaching Hospitals NHS Trust, UK; Microbiology and Pharmacy Department, York and Scarborough Teaching Hospitals NHS Trust, UK; Microbiology and Pharmacy Department, York and Scarborough Teaching Hospitals NHS Trust, UK; Microbiology and Pharmacy Department, York and Scarborough Teaching Hospitals NHS Trust, UK; Microbiology and Pharmacy Department, York and Scarborough Teaching Hospitals NHS Trust, UK; Microbiology and Pharmacy Department, York and Scarborough Teaching Hospitals NHS Trust, UK; Microbiology and Pharmacy Department, York and Scarborough Teaching Hospitals NHS Trust, UK; Microbiology and Pharmacy Department, York and Scarborough Teaching Hospitals NHS Trust, UK; Microbiology and Pharmacy Department, York and Scarborough Teaching Hospitals NHS Trust, UK

## Abstract

**Background:**

Increasing antimicrobial resistance is a global threat, highlighting the need for robust antimicrobial stewardship (AMS) practices. Pharmacists and pharmacy technicians are integral members of AMS teams, and joint ward rounds with consultant microbiologists provide an important forum to optimize antibiotic use and improve patient care.

**Objectives:**

To evaluate the impact of consultant microbiologist– and pharmacist–led AMS ward rounds at York and Scarborough hospitals over five months, focusing on the number of patients reviewed, recommendations made, uptake of these recommendations, and savings achieved through timely IV-to-oral switches (IVOS) and antibiotic stops directly driven by AMS advice.

**Methods:**

Twice-weekly AMS ward rounds were conducted jointly by an antimicrobial pharmacist, antimicrobial pharmacy technician, and a consultant microbiologist from October 2025 to February 2026. Patients started on broad-spectrum antibiotics by the medical and surgical teams were identified via the Trust electronic prescribing system by AMS pharmacy technicians. Data were retrospectively collected on the number of patients reviewed, antibiotic recommendations (de-escalation, escalation, continuation/review, or stop date), uptake of recommendations, referrals to outpatient parenteral antimicrobial therapy (OPAT), nursing time saved, and estimated financial savings associated with IV-to-oral switches or antibiotic stops enabling timely discharge. Two patient cohorts were included: those receiving piperacillin/tazobactam and patients receiving other broad-spectrum antibiotics as identified by the clinical team.

**Results:**

Over five months, 78 AMS ward rounds were conducted, reviewing 591 patients across 686 patient visits, as some patients were reviewed more than once. Of the patients reviewed, 489 received piperacillin/tazobactam and 197 received alternative broad-spectrum antibiotics. A total of 750 recommendations were made, of which 668 (89.1%) were actioned. Recommendations included de-escalation in 105 cases (14.0%), of which 84 (80.0%) were actioned and 21 (20.0%) were not. Stop dates were recommended for 157 cases (20.9%), with 140 (89.2%) implemented and 17 (10.8%) not actioned. Continuation or review of therapy was advised in 217 cases (28.9%), with all 217 (100%) implemented. Escalation was recommended in 15 cases (2.0%), with 14 (93.3%) actioned and one (6.7%) not implemented. Antibiotic switches based on culture or organism results occurred in 37 cases (4.9%), with 34 (91.9%) actioned and three (8.1%) not actioned. IV-to-oral switches were recommended in 207 cases (27.6%), with 170 (82.1%) implemented and 37 (17.9%) not implemented. Twelve patients (1.6%) were referred to OPAT, of which nine (75.0%) were accepted and three (25.0%) were not actioned. These interventions resulted in an estimated saving of 1896 h of Band 6 nursing time through reduced IV antibiotic administration. Estimated financial savings from IV-to-oral switches and antibiotic stops directly driven by AMS recommendations totalled £53 802.96.

**Conclusions:**

Consultant microbiologist– and pharmacist–led AMS ward rounds resulted in high implementation of stewardship recommendations (89.1%), while delivering measurable workforce and financial benefits, supporting their role as an effective, sustainable and measurable antimicrobial stewardship intervention in acute care.

